# Genome sequence and organization of the *Mythimna* (formerly *Pseudaletia) unipuncta* granulovirus Hawaiian strain

**DOI:** 10.1038/s41598-020-80117-3

**Published:** 2021-01-11

**Authors:** Yinü Li, Xingjian Liu, Ping Tang, Huan Zhang, Qilian Qin, Zhifang Zhang

**Affiliations:** 1 Biotechnology Research Institute, Chinese Academy of Agricultural Sciences, Beijing, China; 2 Institute of Zoology, Chinese Academy of Sciences, Beijing, China; 3 Jiangsu University of Science and Technology, Zhenjiang, China

**Keywords:** Systems virology, Sequence annotation

## Abstract

Purified occlusion bodies (OBs) of *Mythimna *(formerly *Pseudaletia*) *unipuncta* (the true armyworm) granulovirus Hawaiian strain (MyunGV-A) were observed, showing typical GV morphological characteristics under scanning and transmission electron microscopy (EM). The genome of MyunGV-A was completely sequenced and analysed. The genome is 176,677 bp in size, with a G+C content of 39.79%. It contains 183 open reading frames (ORFs) encoding 50 or more amino acids with minimal overlap. Comparison of MyunGV-A with TnGV, XcGV, and HearGV genomes revealed extensive sequence similarity and collinearity, and the four genomes contain the same nine homologous regions (*hrs*) with conserved structures and locations. Three unique genes, 12 baculovirus repeated ORF (bro), 2 helicase, and 3 enhancin genes, were identified. In particular, two repeated genes (ORF39 and 49) are present in the genome, in reverse and complementarily orientations. Twenty-four OB proteins were identified from the putative protein database of MyunGV-A. In addition, MyunGV-A belongs to the *Betabaculovirus* group and is most closely related to TnGV (99% amino acid identity) according to a phylogenetic tree based on the combined amino acid sequences of 38 core gene contents.

## Introduction

Baculoviruses are a large family of rod-shaped, invertebrate-infecting viruses with large circular, covalently closed, double-stranded DNA genomes of between 80 and 180 kb. This family was initially taxonomically subdivided into nucleopolyhedroviruses (NPVs) or granuloviruses (GVs) based on viral occlusion morphology^1^. However, when an increasing number of genome sequences became available, it was clear that lepidopteran NPVs and GVs are more closely related to each other than to dipteran and hymenopteran NPVs. Therefore, a new taxonomic division that follows the evolution of the host more closely^2^ was accepted by the International Committee on Taxonomy of Virus (ICTV). In the 10th report of the ICTV (online, 2019), the family *Baculoviridae* was still divided into four genera: *Alphabaculovirus*, *Betabaculovirus*, *Deltabaculovirus* and *Gammabaculovirus* (https://talk.ictvonline.org/ictv-reports/ictv_online_report/). To date, 85 baculovirus genomes have been sequenced (http://www.ncbi.nlm.nih.gov/genomes/GenomesGroup.cgi?opt=virus&taxid=10442), including 55 from *Alphabaculovirus* (lepidopteran NPVs), 26 from *Betabaculovirus* (lepidopteran GVs), 1 from *Deltabaculovirus* (dipteran NPVs) and 3 from *Gammabaculovirus* (hymenopteran NPVs).

Betabaculoviruses are granuloviruses (GVs) infecting only lepidopteran hosts, whereas alphabaculoviruses, deltabaculoviruses and gammabaculoviruses are nucleopolyhedroviruses (NPVs) isolated from a wider range of hosts, including lepidopterans, dipterans and hymenopterans.

Lepidopteran NPVs are further divided into two groups, I and II, based on gene content^3^. Notably, the budded virus (BV) fusion protein in Group I NPVs is GP64, whereas Group II NPVs lack *gp64* and utilize the F protein^4^. GVs are classified into three types according to tissue tropism^5^. Type I GVs, such as Xestia c-nigrum GV (XcGV), kill hosts at a slow speed by only infecting the midgut epithelium and fat body tissue^6^. Type II GVs, such as *Cydia pomonella* GV (CpGV), kill hosts at a rapid speed, similar to typical lepidopteran NPVs, by infecting most of the host’s major tissues^7^. Type III GVs infect only the midgut epithelium. Only one GV, *Harrisina brillians* GV (HabrGV)^8^, has been identified as Type III. Phylogenetic analysis on the basis of conserved genes of GVs does not show certain monophyletic origins for these different types of pathogenesis^9^.

*Mythimna unipuncta* granulovirus (MyunGV-A), originally described as *Pseudaletia unipuncta* granulovirus (PsunGV) based on an isolated Hawaiian population of *Mythimna* (*Pseudaletia*) *unipuncta*^10^, was identified as PsunGV by the ICTV in 2002. Until 2017, PsunGV was proposed to be renamed MyunGV-A by the ICTV to reflect the fact that the new species MyunGV-B is the second distinct betabaculovirus to be isolated from the host *Mythimna* (*Pseudoletia*) *unipuncta*.

MyunGV-A (PsunGV-H) was first discovered by synergistic factors (described later as enhancin)^10^. Subsequent studies on MyunGV-A mostly focused on the mechanisms of enhancement and the enhancin gene. The enhancin of MyunGV-A can interact with viral particles and increase the binding of viral particles to insect midgut microvilli, thereby dramatically promoting the oral infectivity of *Mythimna unipuncta* NPV and decreasing the larval survival time^11^. The enhancin of MyunGV-A comprising 901 amino acids have been purified and characterized^12^. Overall, high-throughput sequencing of baculovirus genomes appears to be essential for analysing the molecular mechanisms of baculovirus infection and understanding baculovirus genome evolution. In this study, the morphological characteristics of MyunGV-A were observed by electron microscopy (EM). We present the complete sequence and organization of the MyunGV-A genome and compare it with other baculoviruses by genomic and phylogenetic analysis. A total of 24 OB proteins of MyunGV-A were identified.

## Materials and methods

### Virus preparation and DNA extraction

MyunGV-A (PsunGV-H) was obtained from Tanada Y. and kept at the Institute of Zoology, Chinese Academy of Sciences^13^. The virus was propagated in laboratory stocks of healthy second-instar *M. separate* larvae by per os infection. The occlusion bodies (OBs) produced in larval cadavers were purified by a standard method^14^.

To extract viral DNA, the purified OBs were resuspended in 0.1 M sodium carbonate solution [0.1 M Na_2_CO_3_, 0.17 M NaCl, 0.01 M EDTA (pH 10.5)] and incubated at 37 °C for 1 h. The pH was adjusted to 7.0 with 0.1 M HCl. Sarcosyl 0.5% and proteinase K 0.25 mg/mL were added to the sample and incubated at 37 °C for 2 h and 65 °C for 2 h. Genomic DNA was extracted with an equal volume of phenol and chloroform. The DNA was precipitated with two volumes of 100% ethanol, washed with 70% ethanol, and dissolved in TE buffer [10 mM Tris–HCl (pH 8); 1 mM EDTA].

### Electron microscopy observation

OBs of MyunGV-A were observed by scanning electron microscopy (SEM; Hitachi S3400N) and transmission electron microscopy (TEMl; JEOL JEM1230) according to standard methods^15^.

### DNA sequencing and analysis

A random genomic library of MyunGV-A was constructed according to the “partial filling-in” method^16^. A total of 831 recombinant plasmids containing 1.5 to 5.0 kb viral DNA fragments were prepared for sequencing using a BigDye Terminator v3.1 (ABI) and a 3130XL Genetic analyser (ABI). The combined sequence generated from these clones represented sixfold genomic coverage. The gaps and ambiguities in the assembled sequence were resolved by PCR. All sequences were assembled into contigs using SeqMan from the DNASTAR 7.0 software package.

ORFs were defined using ORF Finder (http://www.ncbi.nlm.nih.gov/gorf/gorf.html). The criterion for defining an ORF was a size of 50 or more codons with minimal overlap. DNA and protein comparisons were performed using BLAST (http://blast.ncbi.nlm.nih.gov/Blast.cgi). For protein homology detection, we used the HHpred webserver for the translated ORFs^17,18^. Multiple alignments and percentage identities were obtained using ClustalW. Promoter motifs present upstream of the putative ORFs were screened as described previously^19^. Identity among homologous genes was determined with MegAlign software using ClustalW with default parameters. Homologous repeat regions (*hrs*) were analysed by Tandem Repeats Finder (https://tandem.bu.edu/trf/trf.html). GeneParityPlot analysis was performed as described by Hu et al.^20^.

### Protein analysis of OBs of MyunGV-A

Fresh purified OBs of MyunGV-A suspended in ddH_2_O were incubated with an equal volume of lysis buffer (0.1 M Na_2_CO_3_, 0.17 M NaCl, 0.01 M EDTA, pH 10.6) at 4 °C for 1 h. The pH was adjusted to 8.0 with 0.1 M HCl. The samples were added to 10 mM Tris–HCl containing β-mercaptoethanol (0.2%) and sodium dodecyl sulfate (SDS) at 95 °C for 10 min. The proteins of MyunGV-An OBs were separated by SDS-PAGE using an 8% to 15% gradient gel. The protein bands were excised into 29 samples according to molecular weight from small to large for LC–MS/MS analysis (LCQ Deca Xp plus, ThermoFinnigan). LC–MS/MS analysis and protein identification were performed as described by Shi XF^21^. The raw files of MS spectra were searched against the putative protein database of MyunGV-A (NC_013772.1).

### Phylogenetic analysis of MyunGV-A

The amino acid sequences encoded by the 38 core genes described for all members of family Baculoviridae^22^ of 82 complete baculovirus genomes (excluding 3 incomplete genomes) in the NCBI genome database (https://www.ncbi.nlm.nih.gov/genomes/GenomesGroup.cgi?opt=virus&taxid=10442) were joined together according to a consistent order (ORF order of AcMNPV) and aligned using MAFFT with default parameters. A phylogenetic tree based on these sequences was constructed using MEGA 7 MEGA 7.0.14^23^. Maximum likelihood (ML) tree construction methods were used with 1000 bootstrap resamples. The GTR + G + I substitution model was used for ML analysis.

## Results and discussion

### Electron microscopy observation

**Figure 1:**
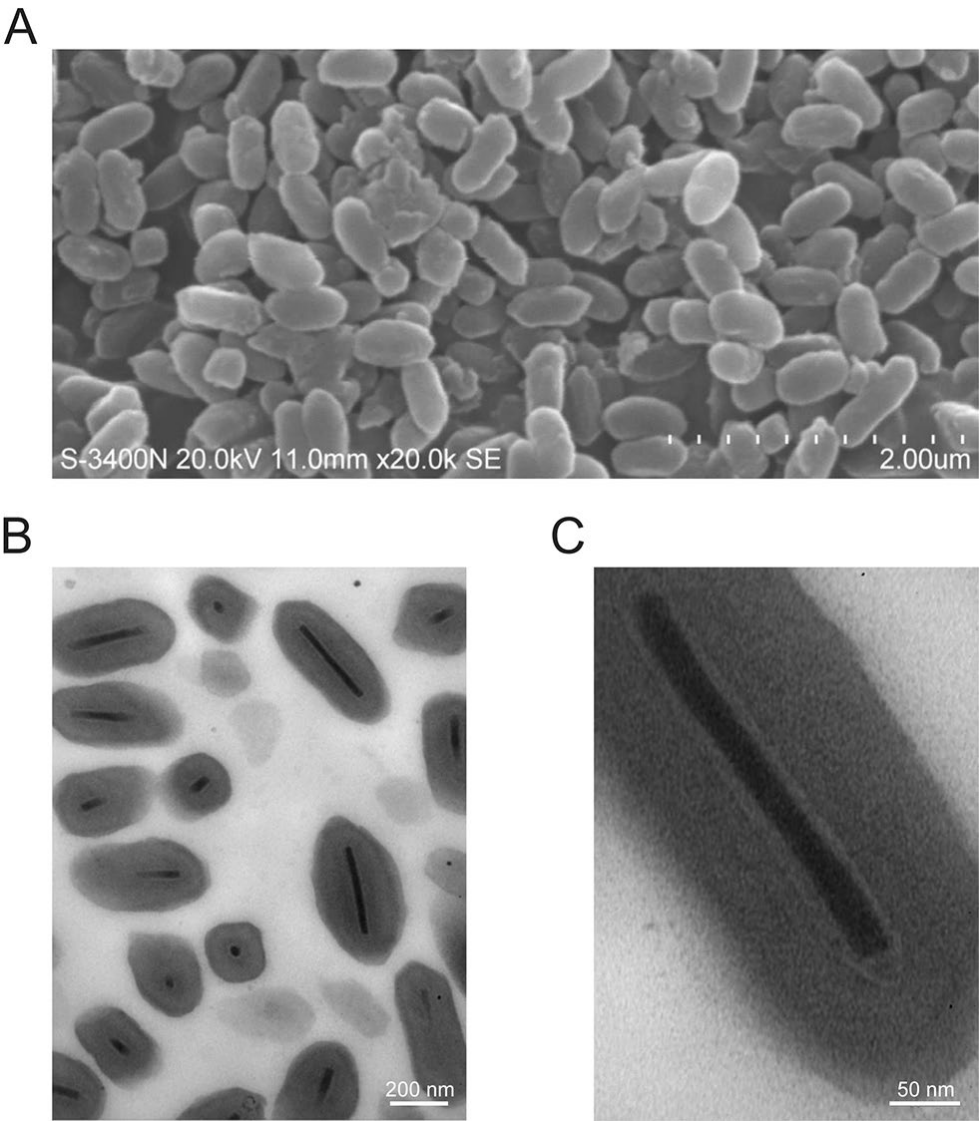
A scanning electron micrograph of MyunGV-A (**A**) and transmission electron micrograph of MyunGV-A (**B**,**C**).

SEM revealed that the purified OBs of MyunGV-A have elongated ellipse shapes, with a length of approximately 0.5 μm and a width of approximately 0.3 μm (Fig. 1A). TEM showed a single rod-shaped ODV of approximately 300 nm in length and 40 nm in width embedded in a granular OB (Fig. 1B,C). These are typical GV morphological characteristics.

### Sequence and genome characteristics of MyunGV-A

The size of the MyunGV-A genome is 176,677 bp (GenBank accession no. NC_013772), with a G+C content of 39.79%. MyunGV-A is the second largest GV sequenced to date, with XcGV (178,733 bp)^6^ being larger. Computer-assisted ORF analysis detected 372 ORFs of 50 or more codons and 9 homologous regions (*hrs*) in the MyunGV-A genome; 189 ORFs overlap significantly or are completely contained within other MyunGV-An ORFs. The deduced protein sequences of these 189 ORFs show no significant homology to protein sequences in GenBank. The remaining 183 ORFs and 9 h are shown in Table 1 according to location, orientation, size of the predicted amino acid sequence, potential baculovirus homologues, best matched baculovirus ORF and BLAST score (bits).

**Table 1:** MyunGV-A (PsunGV-H) open reading frames (ORFs) and homologous repeat regions (*hrs*).

ORF	Name	Position	Length (aa)	Promoter	Homologous ORF#/amino acid identity (%)					
					AcMNPV	XcGV	HearGV	TnGV	MyunGV-B	CpGV
1	Granulin	1>747	248	L	8/55	1/100	1/100	1/99	1/98	1/87
2	1629 capsid	796<1518	240	L	9/38	2/72	2/72	2/98	2/44	2/39
3	pk	1499>2353	284	L	10/33	3/91	3/92	3/100	3/70	3/47
4	Unknown	2399<3274	291	L	–	4/58	4/57	4/99	4/31	–
5	p10	3287>3646	119	L	137/33	5/90	5/92	5/100	5/65	–
6	Unknown	3682<4245	187	E	–	7/90	6/89	6/100	6/73, 7/50	4/35
7	Unknown	4235>4495	86	L	–	8/81	7/80	7/99	8/54	5/21
8	ie-1	4499<5923	474	E	147/27	9/77	8/76	8/100	9/58	7/28
9	Unknown	5946>6533	195	L	146/27	10/86	9/85	9/100	10/58	8/35
10	Unknown	6581<6880	99	L	145/39,150/44	11/97	10/96	10/100	11/83	9/53
11	odv-e18	6889<7140	83	L	143/44	12/90	11/90	–	12/81	14/61
12	p49	7144<8502	452	L	142/32	13/85	12/84	11/99	13/79	15/43
13	Unknown	8576<9265	229	E	–	14/88	13/88	12/99	14/60	–
14	odv-e56	9279<10,340	353	L	148/38	15/83	14/86	13/99	15/73	18/57
15	Unknown	10,370>10,576	68	–	–	16/81	15/78	14/100	16/58	19/35
16	pep	10,613<11,185	190	L	131/26	17/95	16/95	15/100	18/61	20/44
17	pep	11,262<11,723	153	L	–	18/93	17/92	16/99	19/84	23/53
18	pep/p10	11,744<12,907	387	E/L	–	19/94	18/94	17/100	20/80	22/49
19	Unknown	12,992>13,258	88	L	145/26,150/34	105/39,20/36	107/37,19/35	18/100	–	–
20	p94	13,339>15,777	812	E/L	134/36	21/70	20/71	19/99	–	–
21	Unknown	15,834<17,309	491	–	–	22/73	160/45	20/99	137/41	–
22	Unknown	17,390<18,463	357	–	–	23/55	22/56	21/99	23/44	–
23	Unknown	18,582<18,920	112	E/L	–	24/75	23/75	22/100	24/59	–
24	Unknown	20,373>21,686	437	E	–	25/61	24/60	23/99	26/24	29/56
25	Unknown	22,224>23,210	328	E/L	–	26/56	25/57	24/98	28/39	30/22
26	efp	23,278>25,023	581	L	23/20	27/80	26/79	25/99	29/52	31/32
27	Unknown	25,152>25,628	158	E	–	28/25	27/27	–	–	–
28	Unknown	26,027<26,737	236	L	–	29/80	28/78	26/100	31/55	33/27
29	Unknown	26,756<27,334	192	L	–	30/81	29/81	27/99	32/72	34/47
30	pif-3	27,364>27,948	194	L	115/41	32/91	30/92	28/100	33/66	35/46
31	Unknown	27,955>28,287	110	L	–	33/53	31/52	–	–	–
32	Unknown	28,313>28,654	113	L	–	34/99	32/99	29/100	35/85	39/49
33	lef-2	28,656>29,240	194	E/L	6/23	35/82	33/81	30/97	36/57	41/40
34	Cp35Ra	29,244>29,513	89	L	–	36/87	34/87	31/100	37/43	42/36
35	Unknown	29,549<29,959	136	L	–	–	–	32/100	–	–
36	Unknown	30,049<30,369	106	E	–	38/89	36/86	–	38/60	–
37	Unknown	30,494<30,952	152	E/L	–	39/75	37/74	33/100	39/45	45/20
38	mp-nase	31,036<32,817	593	L	–	40/81	38/77	34/99	40/53	46/31
39	Unknown	33,277>34,992	571	E	–	–	53/86,157/85	43/97	–	–
40	Unknown	35,052<36,023	323	–	–	42/60	39/58	–	41/25	–
41	p13	36,074>36,910	278	E/L	–	43/91	40/91	35/100	42/66	47/50
42	Unknown	37,028>37,621	197	E/L	–	44/59	41/61	36/100	–	–
43	pif-2	37,638>38,804	388	L	22/51	45/93	42/93	37/100	43/82	48/55
*hr*1	–	38,812–39,431	–	–	–	–	–	–	–	–
44	Unknown	39,435<39,677	80	L	–	46/76	43/71	38/99	44/38	49/33
45	Unknown	39,702>43,343	1213	L	–	47/82,48/51	44/52	39/99	45/43	50/29
46	Unknown	42,743<43,168	141	–	–	49/48	–	40/100	–	–
47	Unknown	43,330<44,112	260	L	106/44	50/84	45/83	41/100	46/68	52/53
48	pif-7	44,157>44,321	54	–	110/27	51/90	46/90	42/100	47/75	53/40
49	Unknown	44,456<46,171	571	E	–	–	53/86,157/85	43/97	–	–
50	Ubiquitin	46,360<46,593	77	L	35/76	52/96	47/96	44/100	48/95	54/82
51	odv-ec43	46,695>47,756	353	L	109/30	53/83	48/83	45/99	50/67	55/44
52	Unknown	47,783>48,115	110	L	108/37	54/92	49/94	46/100	51/76	56/29
53	39 k	48,181<49,077	298	L	36/25	55/89	50/90	47/100	52/63	57/24
54	lef-11	49,058<49,345	95	E	37/28	56/89	51/87	–	53/78	58/53
*hr*2	–	49,441–49,811	–	–	–	–	–	–	–	–
55	Unknown	49,955>50,803	282	E/L		57/72	52/71	48/100	57/55	–
56	*bro*-a/*bro*-f	50,984<52,480	498	E		60/28,131/30	54/31	49/100	–	63/29
57	Unknown	52,710<52,922	70	E		63/47	–	50/98	–	–
58	Unknown	53,074>53,775	233	E/L		151/50	–	–	64/74	–
59	Unknown	53,865>54,401	178	–	–	–	–	–	–	64/34
60	Unknown	54,541<56,619	692	E/L	–	64/64	59/64	51/64	–	–
61	Unknown	56,674<58,119	481	–	–	65/80	60/81	52/71	–	–
62	*bro*	58,220<58,879	219	E/L	2/31	62/45	58/44	53/40	21/42	–
63	he65	59,144<60,775	543	E	105/37	67/82	62/81	54/99	–	–
64	sod	60,936<61,397	153	L	31/57	68/89	63/89	55/99	58/74	59/59
65	cath	61,441<62,457	338	L	127/46	58/59	–	56/100	–	11/54
66	*bro*-a/*bro*-f	62,525<64,015	496	E	2/35	60/40,131/43	55/45	57/95	21/43	–
67	Unknown	64,111<64,281	56	L	–	70/73	64/73	58/100	–	–
*hr*3	–	64,322–64,816	–	–	–	–	–	–	–	–
68	Unknown	64,845<65,033	62	–	–	–	–	59/100	–	–
69	Unknown	65,205<65,459	84	E	–	–	–	60/94	–	–
70	Unknown	65,594>66,247	217	E	–	71/63	65/62	61/98	60/37	–
71	Unknown	66,293>67,327	344	E/L	–	72/79	66/79	62/99	–	–
72	Unknown	67,465>68,862	465	L	–	73/82	67/79	63/99	61/48	–
73	Unknown	68,948<70,021	357	L	–	74/69	68/70	64/99	65/40, 99/29	–
74	Unknown	70,125>70,412	95	L	79/38	75/87	69/86	65/100	66/63	65/36
*hr*4	–	70,464–71,122	–	–	–	–	–	–	–	–
75	Unknown	72,110<72,367	85	–	–	–	70/77	66/98	–	–
76	*bro*-b	72,366>73,178	270	E	2/23	76/84	71/83	67/99	67/61	–
77	p74	73,217>75,349	710	L	138/35	77/89	72/89	68/99	68/71	60/42
78	Unknown	75,346<75,681	111	L	–	–	73/68	69/99	–	–
79	p47	75,764>76,948	394	E	40/42	78/91	74/91	70/100	70/74	68/57
80	Rep-like	76,992<77,480	162	E/L	–	–	75/65	71/100	–	–
81	Rep-like	77,574<77,780	68	E	–	–	76/57	72/100	–	–
82	Unknown	78,087>78,764	225	L	38/42	79/97	77/97	73/100	71/85	69/64
83	p24 capsid	78,786>79,301	171	L	129/23	80/88	78/88	74/100	72/70	71/51
84	p38.7	79,338<79,916	192	E/L	13/21	81/73	79/76	75/99	73/46	73/42
85	lef-1	79,917<80,633	238	E	14/31	82/87	80/87	76/99	74/78	74/50
86	p10	80,710>81,291	193	L	–	83/79	81/77	77/100	75/51	–
87	pif-1	81,311>82,936	541	L	119/35	84/86	82/87	78/100	76/68	75/48
88	fgf-1	82,962<83,663	233	L	–	85/68	83/67	79/99	77/45	76/36
89	Unknown	83,713<84,060	115	E/L	–	86/71	84/71	–	78/43	–
90	Unknown	84,175>84,669	164	E/L	150/38	87/73	85/76	80/100	79/30, 80/59	79/29
91	lef-6	84,673<84,972	99	–	28/29	88/84	86/83	81/100	81/57	80/42
92	dbp	85,033<85,866	277	E	25/23	89/84	87/85	82/100	82/56	81/25
93	Unknown	85,972<86,184	70	E/L	–	–	88/82	83/100	83/61	–
94	Unknown	86,154<86,897	247	–	–	90/73	89/75	84/100	84/52	82/35
95	p45	86,896>88,014	372	L	103/36	91/95	90/96	85/100	85/82	83/55
96	p12	88,043>88,408	121	L	102/21	92/80	91/78	86/99	86/59	84/39
97	p40	88,460>89,572	370	E/L	101/20	93/91	92/92	87/100	87/76	85/50
98	p6.9	89,629>89,811	60	L	100/–	94/93	93/90	–	88/79	86/56
99	lef-5	89,847<90,647	266	L	99/39	95/90	94/89	88/100	89/74	87/56
100	38 K	90,570>91,481	303	L	98/37	96/84	95/85	89/99	90/67	88/48
101	Unknown	91,502<91,975	157	L	96/32	97/92	96/92	90/100	91/82	89/49
102	Helicase-1	91,974>95,450	1158	L	95/26	98/89	97/88	91/100	92/78	90/37
103	odv-e25	95,536<96,195	219	L	94/36	99/96	98/95	94/100	93/84	91/65
104	Unknown	96,234<96,710	158	L	93/33	100/94	99/96	95/99	94/66	92/40
105	p33	96,821>97,576	251	L	92/36	101/95	100/94	96/100	95/82	93/54
106	ChaB	97,582<97,845	87	L	60/67	102/95	103/82	97/100	96/80	–
107	Unknown	97,880<98,107	75	E/L	–	–	104/74	98/98	–	–
108	Chitinase	98,275>100,032	585	E/L	126/62	103/87	105/87	99/100	–	10/60
109	Unknown	100,414>100,755	113	E/L	–	106/68	108/70	100/100	–	–
110	gp37	100,824>101,570	248	E/L	64/45	107/82	109/83	101/99	–	13/44
111	Unknown	101,679>102,152	157	E	–	108/78	110/84	102/100	–	–
112	*bro*-c	102,340>103,416	358	E	2/20	109/76	101/68	103/98	–	–
113	Unknown	103,455>103,679	74	–	–	–	–	–	–	–
114	lef-4	103,733<105,091	452	–	90/31	110/86	112/86	104/99	100/63	95/42
115	p39 capsid	105,143>106,126	327	L	89/28	111/80	113/80	105/99	101/77	96/38
116	odv-ec27	106,272>107,138	288	L	144/29	112/94	114/95	106/100	102/81	97/45
117	Unknown	107,471<108,685	404	E	–	113/81	116/81	107/100	103/54	99/27
*hr*5	–	108,728–109,096	–	–	–	–	–	–	–	–
118	*bro*-d	109,118<110,389	423	–	2/23	114/84	117/86	108/100	104/68	–
119	Unknown	110,368>111,543	391	–	–	115/80	118/79	109/100	105/45	–
120	Unknown	111,652>112,023	123	E/L	–	116/92	119/92	110/100	106/66	100/42
121	Unknown	112,066<112,614	182	E	–	117/73	120/73	111/100	107/39	–
122	vp91	112,702<114,957	751	L	83/29	118/79	121/80	112/99	108/58	101/35
*hr*5a	–	113,797–113,923	–	–	–	–	–	–	–	–
123	tlp20	114,923>115,417	164	L	82/23	119/85	122/86	113/100	109/63	102/25
124	Unknown	115,438>116,001	187	L	81/47	120/96	123/96	114/100	110/80	103/57
125	gp41	116,058>116,930	290	E/L	80/32	121/91	124/91	115/100	111/71	104/51
126	Unknown	117,000>117,323	107	L	78/25	122/84	125/84	116/100	112/52	105/29
127	vlf-1	117,307>118,422	371	E/L	77/32	123/89	126/89	117/100	113/75	106/55
128	Unknown	118,437<118,973	178	E	–	124/84	127/84	118/100	114/64	–
129	Unknown	119,015>119,272	85	L	76/34	125/97	128/98	119/100	115/88	107/58
130	Unknown	119,344>119,781	145	E/L	75/29	126/93	129/93	120/100	116/54	108/35
131	Unknown	119,833>120,150	105	L	150/29	20/33,105/31	130/36	121/99	–	–
132	Unknown	120,192<120,629	145	E	–	128/71	131/71	122/100	–	–
133	Unknown	120,702<121,781	359	–	–	–	–	–	–	–
134	lef-7	121,991>122,959	322	E	–	129/56	132/57	123/95	–	–
135	*bro*-a/f	123,207>123,932	241	E	2/30	60/56,131/50	133/51,54/47	127/81	21/51	–
136	*bro*-a/f	124,050>124,505	151	E/L	–	60/30,131/34	54/69,133/30	125/58	–	–
137	*bro*-a/f	124,555>126,084	509	E/L	2/27	60/70,131/49	54/67	127/82	21/48	–
138	dna pol	126,186<129,470	1094	E	65/34	132/86	134/86	128/99	117/75	111/52
139	Desmoplakin	129,469>131,460	663	–	66/29	133/68	135/68	129/99	118/47	112/50
140	lef-3	131,553<132,590	345	E	67/28	134/64	136/65	130/99	119/48	113/25
141	pif-6	132,559>132,969	136	–	68/34	135/91	137/91	131/100	120/79	114/44
142	Unknown	133,028>133,543	171	–	–	136/71	138/70	132/100	121/43	115/33
143	iap	133,631>134,503	290	–	–	137/82	139/81	133/99	122/53	116/31
144	Unknown	134,625>136,646	673	E/L	–	138/26	–	134/99	–	–
145	lef-9	136,758>138,251	497	–	62/56	139/93	140/93	135/100	123/81	117/62
146	fp	138,304>138,516	70	E/L	61/31	140/91	141/92	136/100	124/83	118/45
147	Unknown	138,550>138,702	50	L	–	–	–	137/100	–	–
148	DNA ligase	138,757<140,415	552	E	–	141/89	142/89	138/100	125/71	120/43
149	Unknown	140,592>140,831	79	E	–	142/81	143/80	139/100	126/62	121/27
150	Unknown	140,892>141,092	66	–	–	143/95	144/95	140/100	127/81	122/56
151	fgf	141,155<142,369	404	E	32/27	144/74	145/73	141/99	128/46	123/28
152	alk-exo	142,339>143,760	473	–	133/34	145/78	146/77	142/100	129/61	125/41
153	Helicase-2	143,830>145,203	457	L	–	146/85	147/87	143/99	130/71	126/49
154	Unknown	145,314>146,315	333	E	112/30,113/40	147/80	148/79	144/99	131/61	–
155	lef-8	146,356<148,938	860	L	50/48	148/93	149/93	145/99	132/79	131/61
156	odv-e66	149,009<151,012	667	L	46/40	149/92	150/92	146/99	133/79	37/44
*hr*6	–	151,030–151,402	–	–	–	–	–	–	–	–
157	Enhancin-1	151,411<153,897	828	L	–	150/74	151/74	147/99	134/47	–
158	*bro*-f	154,072<155,493	473	E	–	131/66	133/67	148/94	21/67	–
159	Enhancin-3	155,673>158,378	901	L	–	154/80	153/80	149/99	135/35	–
160	Unknown	158,419>159,585	388	L	–	155/89	154/90	150/99	–	–
161	Unknown	159,833>160,012	59	–	–	157/94	155/91	151/96	–	–
162	*bro*-g	160,230<161,075	281	–	2/27	159/56	158/50	–	–	–
163	Unknown	161,223<161,435	70	E	111/42	160/70	–	152/86	136/63	–
164	Unknown	161,623>163,113	496	E/L	–	161/77	160/76	153/100	137/51	–
165	Unknown	163,143<163,706	187	E	–	162/72	161/72	154/97	138/40	–
*hr*7	–	163,796–164,430	–	–	–	–	–	–	–	–
166	Unknown	164,387>164,587	66	–	–	–	–	–	–	–
167	*bro*-g	164,594>165,649	351	–	–	159/64	159/82	155/92	–	–
168	Unknown	165,772<165,939	55	–	–	–	162/83	156/100	139/56	
169	Unknown	165,865>166,221	118	–	–	165/91	163/94	157/100	–	132/35
170	Enhancin-4	166,267<168,840	857	L	–	166/78	164/79	158/99	140/55	–
171	Unknown	169,109>169,438	109	L	–	167/71	165/71	159/98	141/55	
*hr*8	–	169,581–169,956	–	–	–	–	–	–	–	–
172	Unknown	169,958<170,146	62	E	–	170/87	168/87	161/100	142/38	133/53
173	Unknown	170,133>170,552	139	–	53/35	171/88	169/87	162/100	143/56	134/42
174	Unknown	170,556<171,677	373	L	–	172/71	170/72	163/100	144/50	135/43
175	Unknown	171,705<171,908	67	L	–	173/86	171/86	164/100	145/72	–
176	lef-10	171,886>172,098	70	L	53a/31	174/95	172/94	165/100	146/78	137/34
177	vp1054	171,977>172,948	323	–	54/31	175/92	173/91	166/100	147/78	138/49
178	Unknown	173,035>173,217	60	E/L	–	176/91	174/91	167/100	148/72	–
179	Unknown	173,331>173,666	111	E	–	177/73	175/72	168/100	149/42	–
180	fgf-1	173,710>174,633	307	–	–	178/69	176/68	169/98	150/52	140/29
181	Unknown	174,743>175,375	210	E	–	179/75	177/75	170/100	151/45	–
182	me53	175,416>176,330	304	E	139/20	180/88	178/87	171/100	152/63	143/34
183	Unknown	176,336>176,659	107	–	–	181/85	179/84	172/100	153/63	–

Putative MyunGV-A ORFs are listed in column 1 along with the gene homologues designated in column 2. Column 3 indicates ORF location and transcriptional direction on the MyunGV-A genome. Column 4 indicates the number of amino acids. Column 5 indicates the presences of early (E) and/or late (L) promoters located upstream of start codon of each ORF. E indicates a TATA sequence followed by a CAGT or CATT mRNA start site sequence 20–40 nucleotides downstream, with 180 bp upstream of the start codon. L indicates the presence of a (A/T/G)TAAG sequence. Column 6–11 list the homologous ORF and percent of amino acid identity from AcMNPV, XcGV, HearGV, TnGV, MyunGV-B and CpGV respectively.

The first nucleotide of the granulin start codon was defined as nucleotide 1, and the ORF encoding granulin was accordingly designated as the first ORF. The putative ORFs were numbered sequentially in this orientation. Ninety-nine ORFs are in the granulin-sense orientation and 84 in the opposite orientation. A total of 183 putative ORFs of MyunGV-A were searched for promotor motifs at 180 bp upstream of the initiation codon of each ORF; only 42 were found to have a canonical baculovirus early gene promoter motif (a TATA box followed by a CAGT or CATT motif 20 to 40 bp downstream)^24,25^. Seventy-five ORFs only possess a late promoter motif ((A/T/G) TAAG); 75 contain both early and late promoter motifs, which might allow transcription during both early and late stages of infection. Thirty-four lack any recognizable canonical promoter motif.

### Comparison of MyunGV-An ORFs to other baculoviruses

Comparison of gene organization and homology between MyunGV-A and other baculovirus genomes provides insight into gene conservation and implications for the diversity of baculoviruses. MyunGV-A shares 88 ORFs with AcMNPV, 166 with XcGV, 169 with HearGV and TnGV, 139 with MyunGV-B and 104 with CpGV (Table 1). The average amino acid sequence identities of homologous ORFs between MyunGV-A and AcMNPV, XcGV, HearGV, TnGV, MyunGV-B and CpGV are 34%, 79%, 79%, 98%, 62% and 44%, respectively. A total of 180 ORFs were assigned a function or are homologous with other baculoviruses, of which three ORFs (68, 69 and 147) have homologues only with TnGV. ORF68 and ORF147 share 100% homology with TnGV but ORF69 94%. In addition, ORF69 has 37% homology with a kind of bacterium, *Zooshikella ganghwensis*. Three ORFs, ORF113, -133 and -166, were identified as unique to MyunGV-A.

### GeneParityPlot analysis

**Figure 2:**
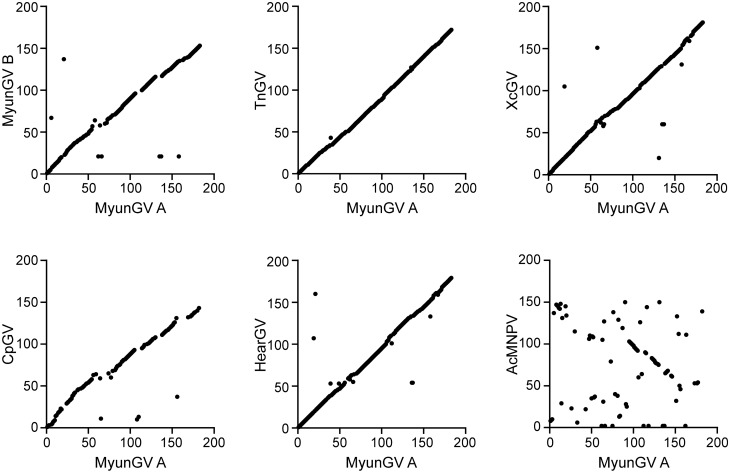
GeneParityPlots analysis.

The gene order of MyunGV-A was compared with that of AcMNPV, XcGV, HearGV, TnGV, MyunGV-B and CpGV by GnenParityPlots analysis (Fig. 2)^20^. The gene organization of MyunGV-A is distinctly different from that of AcMNPV, except for two reverse collinear gene clusters in which one is a 12-gene group including the core gene cluster of four genes, lef-5, 38K(ac98), ac96, and helicase, with relative positions that are conserved in baculovirus genomes^26^. In contrast, the gene order of MyunGV-A exhibits extensive collinearity with XcGV, HearGV, TnGV, MyunGV-B and CpGV, except for several genes in a different order that are almost bro or near bro, with the highest collinearity to TnGV. Interestingly, the arrangement of the MyunGV-A genome shows lower collinearity to MyunGV-B, a virus from the same host, than to XcGV, HearGV and TnGV.

### Homologous regions (hrs)

A typical feature of most baculovirus genomes is the presence of homologous regions (*hrs*) interspersed throughout the genome. The numbers of *hrs* in 82 complete baculovirus genomes range from none to 17, with 12 baculovirus genomes lacking typical *hrs* sequences (Table S1). In general, *hrs* are characterized by AT-rich and imperfect, reiterated palindromic sequences that may be replaced with direct repeats.

Eight major *hr* sequences (*hr1-8*) and one short *hr* sequence (*hr5a*) were identified in the MyunGV-A genome (Table 1). *hr1-8* contains two to five direct imperfect repeats, each of approximately 120 bp, whereas hr5a does not contain multiple repeated sequences. It is interesting to note that *hr5a* is located in ORF122 (vp91), and the same situations exists in the XcGV and HearGV genomes. Six *hrs* were identified in the MyunGV-B genome lacking sequences corresponding to *hr1* and *hr5/5a* of MyunGV-A^27^. No *hrs* were found in the TnGV genome deposited in 2018 (NC_038375.1), and there is no publication on the analysis of the sequence.

**Figure 3:**
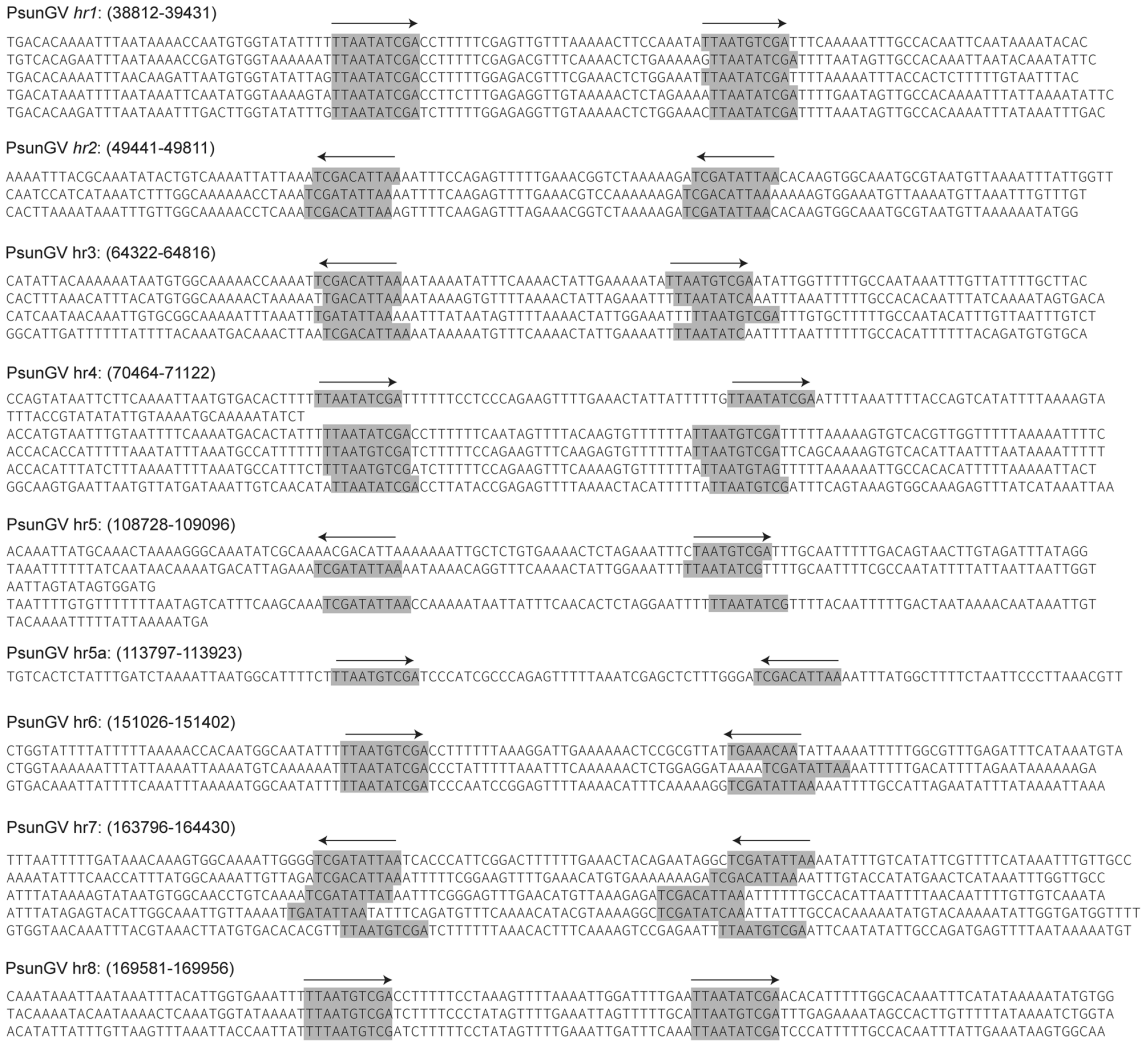
Alignment of homologous regions in the MyunGV-A genome. The conserved 10 bp core sequences (TTAATG/ATCGA) are indicated by shaded boxed. The arrows indicate direction of core sequences.

Although the nucleotide sequences of repeats vary between each *hr*, even in the same *hr*, two highly conserved 10 bp core sequences (TTAAT (G/A) TCGA) were found at the roughly same positions (approximately 35 bp) of each repeat^6^. In the MyunGV-A genome, the core sequences in each repeat of *hr1, -2, -4, -7* and -*8* are in the same directions, while those of *hr3, -5, -5a* and -*6* are in opposite directions (Fig. 3).

*Hrs* have been reported to function in replication origins^28,29^ and serve as enhancers of transcription of early genes^30^. In addition, the number of *hrs* is connected to the replication efficiency or pathogenicity of a baculovirus. Deletion of one to five *hrs* of AcMNPV had little or no effect on virus infection, while deleting six or seven hrs resulted in 90% BV reduction. Deletion of all eight hrs caused 99.9% BV reduction and delay of early and late gene expression but did not completely inhibit virus production^31^.

### Baculovirus repeated ORFs (bro* genes*)

*Bro* genes have been identified in most baculovirus genomes sequenced to date. The number of bro genes in different baculovirus genomes varies considerably. Thirteen of 82 complete baculovirus genomes have only one *bro* gene, though *Lymantria dispar* MNPV (LdMNPV) has 16 *bro* genes. Bro genes are entirely absent from 19 baculovirus genomes sequenced to date (Table S1).

In MyunGV-A, 12 *bro* genes were identified, of which 3 adjacent *bro* genes (ORF135, -136, -137) were found. BLAST results of amino acid sequences of these 3 *bro* genes in NCBI showed that ORF135 best matches with TnGV ORF127 (81%), ORF136 with HearGV ORF54 (69%), and ORF137 with TnGV ORF127 (82%). The TnGV genome have 4 adjacent bro genes (ORF124, -125, -126, -127), and the HearGV genome has 3 pairs of adjacent *bro* genes (hear54 and -55, hear101 and -102, hear158 and -159), but no adjacent *bro* genes were found in the XcGV and MyunGV-B genomes.

The exact function of *bro* genes is not yet clear, though their presence is very significant for baculoviruses. Studies on the function of *bro* genes have mostly focused on BmNPV and have found that BRO-A and C proteins can bind to DNA in infected cells^32^; BRO-A may be involved in influencing host DNA replication, similar to a laminin-binding protein^33^.

In addition, BmNPV BRO proteins act as nucleocytoplasmic shuttling proteins via the CRM1-mediated nuclear export pathway^34^. Recently, BmNPV BRO-B and E proteins associated with host T-cell intracellular antigen 1 homologue (BmTRN-1) were shown to be involved in the inhibitory regulation of certain mRNAs at the post-transcriptional level during infection^35^. The function of other baculovirus BRO proteins has seldom been reported.

### Two repeat genes in MyunGV-A

Two repeat genes (ORF39 and ORF49), with amino acid sequence identities of 100%, were found in the MyunGV-A genome; the former is in the granulin-sense orientation and the latter in the opposite orientation.

There is no homologous gene with these two genes in the XcGV, MyunGV-B and CpGV genomes. Indeed, only one gene, ORF43, of the TnGV genome matches with them, and the amino acid sequence identity is 97%. Two genes, ORF53 and ORF157, in the HearGV genome are homologous, with amino acid sequence identities of 86% and 85%, respectively, and the amino acid sequence identity of ORF53 and ORF157 in the HearGV genome is 99%. One gene with two copies in one baculovirus genome was found in other baculovirus genomes, such as odv-e66, p26 and dbp of EcobNPV^36^ and odv-e66 and p26 of SfMNPV^37^.

BLAST results of amino acid sequences of these two homologous genes in MyunGV-A in NCBI suggested they match *hr3* and *hr4* of *Heliothis virescens* ascovirus 3e (amino acid sequence identities both 49%). In addition, they match the 70.4-kDa C-terminal Zn-finger DNA-binding domain of *Spodoptera frugiperda* ascovirus 1a (amino acid sequence identities of 48%), which suggests that their function may be associated with DNA binding.

### ORFs with no homologues in other baculoviruses

Three ORFs, including ORF113, -133 and -166, were identified as having no homologues in other baculoviruses (Table 1). These three unique ORFs have no recognizable promoter. Protein homology analysis using HHpred showed that GP133 (aa 50–359) is a likely homologue of Mannan-binding lectin serine peptidase 1 (probability, 99.97%; E value, 1.1e-28). Mannan-binding lectin serine peptidase 1 plays a central role in the initiation of the complement lectin pathway^38^. This homology indicates that ORF133 might be related to the complement lectin pathway, which deserves further research. ORF113 encodes an 8.5-kDa protein with one transmembrane domain (aa 5–27, analysed by TMHMM server v2.0) at the N terminus of the protein with no similarity to any proteins in the nonredundant protein database. ORF166 encodes a 7.7-kDa protein with no similarity to any proteins in the nonredundant protein database.

### The large gene in MyunGV-A

In most cases, *helicase* is the largest gene in baculovirus genomes; however, in the MyunGV-A genome, ORF45 encoding 1213 amino acids (longer than *helicase-1*, 1158) is the largest gene. Similar situations are present in the HearGV (ORF44, 1279 aa), TnGV (ORF39, 1213 aa) and MyunGV-B (ORF45, 1507 aa) genomes, though it is divided into two genes, ORF47 and ORF48, in XcGV^6^. Compared with XcGV, the MyunGV-A genome has an additional adenosine (A) at position40315, resulting in a reading frame shift. Protein homology analysis using HHpred and SWISS-MODEL showed no significant similarity to any other known sequences for Myun45.

### Enhancins in MyunGV-A

It was first observed in *Mythimna (formerly Pseudaletia) unipuncta* that GV can increase the rate of infection and fatality of NPV and decrease the larval survival time when GV and NPV coinfect larvae^10^. Subsequent studies found that the factor responsible for synergistic interaction is a GV protein that shows a synergistic effect only when larvae are infected with NPV; it was identified as a synergistic factor (SF)^39^. The synergistic effect of viral enhancing factor (VEF) was also observed in TnGV^40^. The location and sequence of the VEF gene of TnGV have been identified^41^. This enhancing protein (enhancin) can disrupt the midgut peritrophic membrane (PM), thereby resulting in the more efficient passage of virions to host midgut cells^12^. Enhancin was identified as a metalloprotease via the discovery of a zinc-binding site as well as by inhibition with a metal chelator and reactivation with divalent ions^42^.

The MyunGV-A genome has three enhancin genes (Myun157, -159 and -170). Similarly, three enhancin genes were found in MyunGV-B and TnGV, but they show large diversity in amino acid sequence identity compared to MyunGV-A. MyunGV-B enhancins are only 35% to 55% identical to that of MyunGV-A but are as high as 99% identical to that of TnGV. Four enhancin genes were found in the XcGV and HearGV genomes, of which enhancin-1, -3, and -4 have high homology (amino acid sequence identities all above 74%) to three enhancin genes of MyunGV-A. The MyunGV-A enhancin gene (*enhancin-3)* encoding 901 amino acids has been sequenced and characterized^12^. The canonical sequence HEXXH, the zinc-binding site in most metalloproteases, was found in enhancing-3 but not in the other two enhancins. It is not clear why three enhancins are present in MyunGV-A, and the roles of these three enhancins in promoting NPV infection remain unclear.

Enhancins are found mainly in GVs and a few NPVs. They are localized within the granulin matrix in granuloviruses and released to increase virus pathogenicity by acting in the midgut. In contrast, LdMNPV enhancins are located within ODV envelopes and facilitate ODVs to pass the host defence barrier by acting directly on the peritrophic membrane as the nucleocapsids move through the barrier^43^. However, subsequent studies have indicated that LdMNPV enhancins have a function that may assist virus-host cell fusion beyond peritrophic membrane degradation^44^.

### Protein analysis of OBs of MyunGV-A

To date, nine baculovirus proteomic studies have been performed with the intent of revealing infectious mechanisms and virus-host interactions, as follows: six alphabaculviruses—AcMNPV^45,46^, BmNPV^47^, HearSNPV^48^, HearNPV-G4^49^, AgMNPV^50^ and ChchNPV^51^; two betabaculoviruses, ClanGV^52^ and PrGV^53^; and one deltabaculovirus, CuniNPV^54^. In this study, we performed an analysis of MyunGV-An OB proteins. For 29 samples, 24 proteins were identified from the putative protein database of MyunGV-A (NC_013772.1) (Table 2). Among the 24 proteins, 20 were detected with two or more peptides, and the other four were detected with one matching peptide. In addition, 15 of 24 identified proteins were detected in more than one sample. Granulin was found in 28 of the 29 samples (Table S2). The same situations were found for CuniNPV^54^, HearsNPV^48^ and AgMNPV^50^. A noticeable phenomenon was also observed, whereby the identified proteins were not distributed according to their molecular mass in SDS-PAGE gels. The reason was postulated to be incomplete denaturation of OBs and the breakdown of protein complexes or protein processing^54^.

**Table 2:** Analysis of proteins identified from MyunGV-A.

ORF	Protein	ODV of GV		ODV of NPV					Characteristics/function
		PrGV	ClanGV	AcMNPV	HearNPV	AgMNPV	ChchNPV	CuniNPV	
1 (Ac8)	Granulin	1	1	8	1	1	1	1	Occlusion bodies (OBs) matrix protein
11 (Ac143)	ODV-e18	14	13	143	10	139	12	NA	Core gene; Structural protein of ODV envelope
12 (Ac142)	VP49	15	14	142	9	138	11	30	Core gene; a caspase inhibitor; Inhibiting diverse apoptotic stimuli
14 (Ac148)	ODV-e56	16	15	148	15	144	7	102	Core gene; Structural protein of ODV envelope
16 (Cp20)	PEP-1	20	19	NA	120	127	NA	NA	Additional ORFs conserved in GVs ; ORF16L family
17 (Cp23)	PEP-2	22	36	NA	NA	NA	–	NA	Additional ORFs conserved in GVs ; ORF16L family
18 (Cp22)	PEP/P10	21	35	NA	21	133	–	NA	Additional ORFs conserved in GVs ; Similarities to P10 containing a baculovirus PEP C domain
29 (Cp34)	Unknown	29	NA	NA	NA	NA	NA	NA	Unknown
32 (Cp39)	Unknown	-	39	NA	NA	NA	NA	NA	Additional ORFs conserved in GVs
44 (Cp49)	Unknown	NA	NA	NA	NA	NA	NA	NA	Unknown
48 (Ac110)	PIF-7	–	20	–	NA	NA	NA	NA	Unknown
51 (Ac109)	ODV-ec43	46	44	109	94	107	99	69	Core gene; Structural protein of ODV envelope
64 (Ac31)	SOD	–	48	–	106	–	115	NA	Metalloenzyme; Protecting the virus against superoxide radical induced in the environment by sunlight
65 (Ac127)	Cathepsin	–	11	127	–	NA	–	NA	Associated with liquefaction of insects at the end of infection; Promoting the release and spread of progeny virus
67 (Xc70)	Unknown	NA	NA	NA	NA	NA	NA	NA	Unknown
103 (Ac94)	ODV-e25	–	76	94	82	91	86	NA	Core gene; Structural protein of ODV envelope
115 (Ac89)	VP39	81	81	89	78	86	82	24	Core gene; Major capsid protein
120 (Cp100)	Unknown	84	84	NA	NA	NA	NA	NA	Additional ORFs conserved in GVs
125 (Ac80)	GP41	88	89	80	73	79	78	33	Core gene; O-linked glycosylated ODV protein; BV production
156 (Ac40)	ODV-e66	39/44	NA	46	96	114	101	NA	Structural protein of ODV envelope
157 (Xc150)	Enhancin-1	NA	NA	NA	NA	NA	NA	NA	Metalloproteinases; Enhancing the oral infectivity of NPVs
159 (Xc154)	Enhancin-3	NA	NA	NA	NA	NA	NA	NA	Metalloproteinases; Enhancing the oral infectivity of NPVs
174 (Cp135)	Unknown	114	116	NA	NA	NA	NA	NA	Additional ORFs conserved in GVs
175 (Cp136)	Unknown	-	117	NA	NA	NA	NA	NA	Additional ORFs conserved in GVs

Dash (–), the protein was not detected. NA, ORF was not found in the baculovirus genome by BLASTP.

Of the 24 identified proteins, eight are encoded by core genes, including ORF11 (ODV-e18), ORF12 (VP49), ORF14 (ODV-e56), ORF48 (PIF-7), ORF51 (ODV-ec43), ORF103 (ODV-e25), ORF115 (VP39) and ORF125 (GP41); among them, VP39 is the major capsid protein, GP41 is a tegument protein only found in ODVs and is present in the nucleocapsid and the viral envelope as a structural protein of ODVs, and four proteins, including ODV-e18, ODV-e56, ODV-e25 and ODV-ec43, are ODV envelope proteins (Table 2). An ODV envelope protein, ODV-e66, was also identified.

For the 24 identified proteins, six are encoded by additional genes conserved in GVs, including ORF16, ORF17, ORF18, ORF120, ORF174 and ORF175^55^. Among them, proteins encoded by two contiguous ORFs (ORF16 and 17) belong to the CpGV ORF16 L family^56^, and the protein encoded by ORF18 is similar to P10, containing a baculovirus polyhedron envelope protein (PEP) C domain (pfam04513). In addition to structural proteins or those implicated in DNA replication and transcription, four important auxiliary proteins were identified, including SOD, cathepsin and two enhancins. Enhancin-1 and enhancin-3 were detected in our proteomic studies; enhancin-3 was present in 16 samples, while enhancin-1 was present in only 1 sample. Most baculovirus enhancins, including MyunGV-A, are located in the OB matrix, whereas LdMNPV enhancins were found to be associated with ODV envelopes^43,57^. In this study, we did not attempt to determine the specific location of enhancins.

Moreover, four proteins (Myun29, Myun32, Myun44 and Myun67) with unknown functions were detected (Table 2). An increasing number of baculovirus proteomic studies can provide valuable insight into baculovirus structure, infectious mechanisms and interactions with their hosts.

### Phylogenetic analysis of MyunGV-A

**Figure 4:**
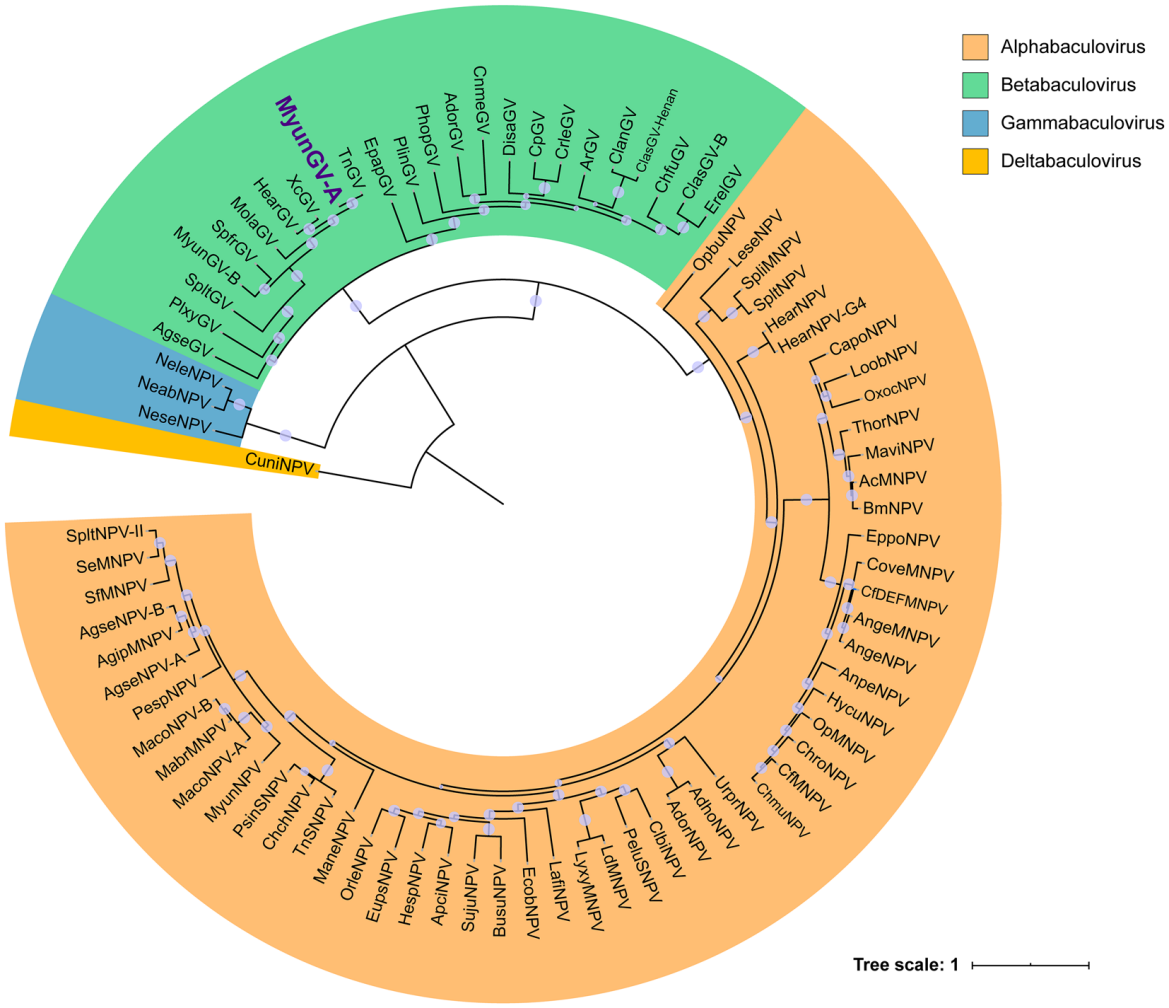
Phylogenetic tree of 82 baculoviruses with complete sequences. The phylogenetic tree was generated using MEGA X^58^ software and performed with the maximum likelihood method and JTT matrix-based model^59^. The result was visualized using iToL^60^.

A phylogenetic tree based on the combined amino acid sequences of 38 core genes from 82 complete baculovirus genomes (Table S1) classified MyunGV-A into clade “a” of *Betabaculovirus,* which clusters infecting the larvae of the Lepidopteran family Noctuidae. Within this clade, MyunGV-A is present into a subcluster together with TnGV, the closest neighbour, sharing a common hypothetical ancestor. XcGV and HearGV form another subcluster next to the MyunGV-A and TnGV subclusters. However, MyunGV-B, another granulovirus from the same host, groups into a subcluster with SpfrGV and slightly away from MyunGV-A across MolaGV (Fig. 4). This is consistent with the above comparison results of gene organization in which MyunGV-A is similar to TnGV, XcGV and HearGV, regardless of genome size, ORF number or gene order.

## Conclusion

The purified OBs of MyunGV-A show typical GV morphological characteristics under EM. The complete MyunGV-A (NC_013772.1) genome is 176,677 bases, with a G+C content of 39.79%, the second largest baculovirus genome to date. It contains 183 ORFs with a minimal size of 50 codons. The genome of MyunGV-A exhibits extensive sequence similarity and collinearity with TnGV, XcGV and HearGV. Three unique genes, 12 bro, 2 helicase and 3 enhancin genes, were identified. In particular, two repeated genes (ORF39 and 49) are present in the genome in reverse and complementarily orientations. Twenty-four OB proteins were identified from the putative protein database of MyunGV-A. According to our phylogenetic tree, MyunGV-A belongs to the *Betabaculovirus* group and is most closely related to TnGV.

## Supplementary Information

Supplementary Tables.
