# Supplementary material for: Genome sequence and organization of the Mythimna (formerly Pseudaletia) unipuncta granulovirus Hawaiian strain
Source: Sci Rep. 2021 Jan 11;11:414. doi: 10.1038/s41598-020-80117-3 (PMC7801670; doi:10.1038/s41598-020-80117-3)
Supplement: Supplementary file 1 — Supplementary Tables. [file 41598_2020_80117_MOESM1_ESM.pdf]

Genome sequence and organization of the *Mythimna* (formerly *Pseudaletia) unipuncta* granulovirus Hawaiian strain

Yinü Li[1†], Xingjian Liu[1†], Ping Tang[3], Huan Zhang[2], Qilian Qin[2*], Zhifang Zhang[1*]

1 Biotechnology Research Institute, Chinese Academy of Agricultural Sciences, Beijing, China; liyinv@caas.cn (Y.L.); liuxingjian@caas.cn (X.L.)
2 Institute of Zoology, Chinese Academy of Sciences, Beijing, China;
3 Jiangsu University of Science and Technology, China;
* Correspondence: bri-zhangzhifang@caas.cn; qinql@ioz.ac.cn.
† These authors contributed equally to this work.

Table S1. Basic information of 85 sequenced baculovirus genomes in GenBank

Genus	Genome	Abbreviation	Accession	Source information	Length	Protein	Neighbors	*hrs*	*bros*
**Alphabaculovirus (55)**	Adoxophyes honmai nucleopolyhedrovirus	AdhoNPV	NC_004690	strain:ADN001	113220	125	-	4	4
	Adoxophyes orana nucleopolyhedrovirus	AdorNPV	NC_011423	isolate:English	111724	121	-	4	3
	Agrotis ipsilon multiple nucleopolyhedrovirus	AgipMNPV	NC_011345	strain:Illinois	155122	163	-	7	5
	Agrotis segetum nucleopolyhedrovirus A	AgseNPV-A	NC_007921		147544	153	-	5	4
	Agrotis segetum nucleopolyhedrovirus B	AgseNPV-B	NC_025960	isolate:English	148981	150	-	6	2
	Antheraea pernyi nucleopolyhedrovirus	AnpeNPV	NC_008035	strain:Liaoning	126629	147	7	3	2
	Anticarsia gemmatalis multiple nucleopolyhedrovirus	AngeMNPV	NC_031761	isolate:AgMNPV-37	131855	156	2	11	6
	Anticarsia gemmatalis nucleopolyhedrovirus	AngeNPV	NC_008520	isolate:AgMNPV-2D	132239	158	-	9	7
	Apocheima cinerarium nucleopolyhedrovirus	ApciNPV	NC_018504		123876	117	-	4	1
	Autographa californica nucleopolyhedrovirus	AcMNPV	NC_001623		133894	156	6	9	1
	Bombyx mori nucleopolyhedrovirus	BmNPV	NC_001962	isolate:T3	128413	143	11	7	5
	Buzura suppressaria nucleopolyhedrovirus	BusuNPV	NC_023442	isolate:Hubei	120420	127	1	0	3
	Catopsilia pomona nucleopolyhedrovirus	CapoNPV	NC_030240	isolate:416	128058	130	-	8	1
	Choristoneura fumiferana DEF multiple nucleopolyhedrovirus	CfDEFMNPV	NC_005137		131160	149	-	13	4
	Choristoneura fumiferana multiple nucleopolyhedrovirus	CfMNPV	NC_004778		129593	146	1	5	1
	Choristoneura murinana nucleopolyhedrovirus	ChmuNPV	NC_023177	strain:Darmstadt	124688	147	-	2	1
	Choristoneura rosaceana alphabaculovirus	ChroNPV	NC_021924	isolate:NB_1	129052	149	-	3	2
	Chrysodeixis chalcites nucleopolyhedrovirus	ChchNPV	NC_007151		149622	151	5	0	4
	Clanis bilineata nucleopolyhedrovirus	ClbiNPV	NC_008293	isolate:DZ1	135454	129	-	0	3
	Condylorrhiza vestigialis MNPV	CoveMNPV	NC_026430		125767	138	-	4	9
	Ectropis obliqua nucleopolyhedrovirus	EcobNPV	NC_008586	strain:A1	131204	126	1	3	2
	Epiphyas postvittana nucleopolyhedrovirus	EppoNPV	NC_003083		118584	136	-	5	1
	Euproctis pseudoconspersa nucleopolyhedrovirus	EupsNPV	NC_012639	strain:Hangzhou	141291	139	-	4	2

Genus	Genome	Abbreviation	Accession	Source information	Length	Protein	Neighbors	*hrs*	*bros*
	Helicoverpa armigera nucleopolyhedrovirus	HearNPV	NC_003094	isolate:C1	130759	137	18	5	3
	Helicoverpa armigera nucleopolyhedrovirus G4	HearNPV-G4	NC_002654		131405	135	18	5	3
	Hemileuca sp. Nucleopolyhedrovirus	HespNPV	NC_021923		140633	137	-	3	2
	Hyphantria cunea nucleopolyhedrovirus	HycuNPV	NC_007767		132959	148	-	6	5
	Lambdina fiscellaria nucleopolyhedrovirus	LafiNPV	NC_026922	isolate:GR15	157977	137	-	2	0
	Leucania separata nucleopolyhedrovirus	LeseNPV	NC_008348	strain:AH1	168041	169	-	8	10
	Lonomia obliqua multiple nucleopolyhedrovirus	LoobNPV	NC_043520		120023	134	-	7	1
	Lymantria dispar multiple nucleopolyhedrovirus	LdMNPV	NC_001973		161046	164	13	13	16
	Lymantria xylina nucleopolyhedrovirus	LyxyMNPV	NC_013953	isolate:LyxyMNPV-5	156344	157	-	13	14
	Malacosoma neustria nucleopolyhedrovirus	ManeNPV	NC_040606	isolate:ManeNPV-T2	130202	131	-	0	2
	Mamestra brassicae multiple nucleopolyhedrovirus	MabrMNPV	NC_023681	strain:K1	152710	159	2	4	6
	Mamestra configurata nucleopolyhedrovirus A	MacoNPV-A	NC_003529	strain:90/2	155060	169	1	4	8
	Mamestra configurata nucleopolyhedrovirus B	MacoNPV-B	NC_004117		158482	168	1	4	7
	Maruca vitrata nucleopolyhedrovirus	MaviNPV	NC_008725		111953	126	-	5	0
	Mythimna unipuncta nucleopolyhedrovirus	MyunNPV	NC_043530		148482	158	-	6	7
	Operophtera brumata nucleopolyhedrovirus	OpbuNPV	NC_040621	isolate:OpbuNPV-MA	119054	130	-	5	2
	Orgyia leucostigma nucleopolyhedrovirus	OrleNPV	NC_010276	isolate:CFS-77	156179	135	-	3	5
	Orgyia pseudotsugata multiple nucleopolyhedrovirus	OpMNPV	NC_001875		131995	152	-	5	3
	Oxyplax ochracea nucleopolyhedrovirus	OxocNPV	NC_043529		113971	124	-	6	0
	Peridroma alphabaculovirus	PespNPV	NC_024625	isolate:GR_167	151109	139	-	2	6
	Perigonia lusca single nucleopolyhedrovirus	PeluSNPV	NC_027923		132831	145	-	2	1
	Pseudoplusia includens SNPV IE	PsinSNPV	NC_026268		139132	141	6	0	2
	Spodoptera exigua multiple nucleopolyhedrovirus	SeMNPV	NC_002169		135611	139	8	6	0
	Spodoptera frugiperda multiple nucleopolyhedrovirus	SfMNPV	NC_009011	isolate:3AP2	131331	143	4	8	1
	Spodoptera littoralis nucleopolyhedrovirus	SpliMNPV	NC_038369	isolate:AN1956	137998	132	1	15	1

Genus	Genome	Abbreviation	Accession	Source information	Length	Protein	Neighbors	*hrs*	*bros*
	Spodoptera litura nucleopolyhedrovirus	SpltNPV	NC_003102	strain:G2	139342	141	-	17	2
	Spodoptera litura nucleopolyhedrovirus II	SpltNPV-II	NC_011616		148634	147	-	7	2
	Sucra jujuba nucleopolyhedrovirus	SujuNPV	NC_028636	isolate:473	135952	131	-	7	4
	Thysanoplusia orichalcea nucleopolyhedrovirus	ThorNPV	NC_019945	isolate:p2	132978	145	1	6	2
	Trichoplusia ni single nucleopolyhedrovirus	TnSNPV	NC_007383		134394	145	-	0	2
	Urbanus proteus nucleopolyhedrovirus	UrprNPV	NC_029997	isolate:Southern Brazil	105555	119	-	0	8
	Wiseana signata nucleopolyhedrovirus **(incomplete)**	WisiSNPV	NC_038370	strain:WisiSNPV	1182	1	-	-	-
**Betabaculovirus (26)**	Adoxophyes orana granulovirus	AdorGV	NC_005038		99657	119	1	0	0
	Agrotis segetum granulovirus	AgseGV	NC_039213	strain:DA	131557	152	2	2	1
	Artogeia rapae granulovirus	ArGV	NC_013797	isolate:Wuhan	108592	120	-	8	0
	Choristoneura fumiferana granulovirus	ChfuGV	NC_008168		104710	116	-	5	0
	Clostera anachoreta granulovirus	ClanGV	NC_015398	isolate:ClanGV-HBHN	101487	123	-	4	0
	Clostera anastomosis granulovirus B	ClasGV-B	NC_038371	ClasGV-B	107439	123	-	0	0
	Clostera anastomosis granulovirus Henan	ClasGV-Henan	NC_022646	isolate:CaLGV-Henan	101818	122	-	1	0
	Cnaphalocrocis medinalis granulovirus	CnmeGV	NC_029304	strain:Enping	111246	118	1	0	3
	Cryptophlebia leucotreta granulovirus	CrleGV	NC_005068	isolate:CV3	110907	128	1	3	0
	Cydia pomonella granulovirus	CpGV	NC_002816	strain:Mexican 1	123500	143	5	0	1
	Diatraea saccharalis granulovirus	DisaGV	NC_028491	strain:Parana-2009; isolate:DisaGV-Parana-2009	98392	125	-	7	0
	Epinotia aporema granulovirus	EpapGV	NC_018875		119082	132	-	16	0
	Erinnyis ello granulovirus	ErelGV	NC_025257	isolate:S86	102759	130	6	5	0
	Harrisina brillians granulovirus **(incomplete)**	HabrGV	NC_038372		1241	3		-	-
	Helicoverpa armigera granulovirus	HearGV	NC_010240		169794	179	-	9	10
	Lacanobia oleracea granulovirus **(incomplete)**	LoGV	NC_038868	substrain:LOGV-S1;	2108	1		-	-

Genus	Genome	Abbreviation	Accession	Source information	Length	Protein	Neighbors	*hrs*	*bros*
				isolate:Scottish					
	Mocis latipes granulovirus	MolaGV	NC_029996	isolate:Southern Brazil	134272	145	-	2	2
	Mythimna (Pseudaletia) unipuncta granulovirus	MyunGV-A	NC_013772	strain:Hawaiin	176677	183	-	9	12
	Mythimna unipuncta granulovirus B	MyunGV-B	NC_033780	isolate:MyunGV#8	144673	153	-	6	5
	Phthorimaea operculella granulovirus	PhopGV	NC_004062		119217	130	1	12	1
	Plodia interpunctella granulovirus	PlinGV	NC_032255	isolate:Cambridge	112536	123	-	7	0
	Plutella xylostella granulovirus	PlxyGV	NC_002593	strain:K1	100999	120	5	4	0
	Spodoptera frugiperda granulovirus	SpfrGV	NC_026511	isolate:VG008	140913	146	1	8	7
	Spodoptera litura granulovirus	SpltGV	NC_009503	isolate:SlGV-K1	124121	136	-	0	6
	Trichoplusia ni granulovirus LBIV-12	TnGV	NC_038375	isolate:LBIV-12	175360	172	-	-	11
	Xestia c-nigrum granulovirus	XcGV	NC_002331		178733	181	-	9	7
**Deltabac ulovirus**	Culex nigripalpus nucleopolyhedrovirus	CuniNPV	NC_003084	isolate:Florida1997	108252	109	-	4	6
**Gammab aculoviru**	Neodiprion abietis NPV	NeabNPV	NC_008252		84264	93	-	5	0
	Neodiprion lecontei nucleopolyhedrovirus	NeleNPV	NC_005906		81755	89	-	0	0
	Neodiprion sertifer nucleopolyhedrovirus	NeseNPV	NC_005905		86462	90	-	6	0

Table S2. List of proteins identified from MyunGV-A

ORF of MyunGV-A	Protein	Peptide Sequence	Length (aa)	Amino acid coverage (%)	Number of times detected in 29 samples	Sequest score (Delta Cn)	Peptides (Hits)
**1**	Granulin	-.FTMQYALAANPDYVAHDVIR.-<br>-.EAQFDPIKDIANQYMVTEDPFRGPGK.-<br>-.IKEFAPDVPLFTGPAY.-<br>-.SLGSVLGDVR.-<br>-.EFAPDVPLFTGPAY.-<br>-.RIQPDTMK.-<br>-.QHDPYYVGPDNR.-<br>-.HNGTTCVIDNK.-<br>-.ERINLSK.-<br>-.EFLRETWTR.-<br>-.ITLFKEIR.-<br>-.DIANQYMVTEDPFRGPGKNVK.-<br>-.DIANQYMVTEDPFR.-<br>-.EAQFDPIKDIANQYMVTEDPFR.-<br>-.LVCNWSGKEFLR.-<br>-.DIANQYMVTEDPFRGPGK.-	248	66.5(165/248)	28	2490.32	249 (249 0 0 0 0)

ORF of MyunGV-A	Protein	Peptide Sequence	Length (aa)	Amino acid coverage (%)	Number of times detected in 29 samples	Sequest score (Delta Cn)	Peptides (Hits)
		-.CYKFTMQYALAANPDYVAHDVIR.-					
		-.EAQFDPIK.-					
		-.PLVYVGTTSAEIEEVMIEVALLFK.-					
11	ODV-e18	-.NNPVVTTATTTR.-<br>-.NMFNPLNNTMR.-	83	27.7(23/83)	5	420.17	42 (42 0 0 0 0)
12	p49	-.VCASDAEGTNTTMLR.-<br>-.QHLASPTDQDLLR.-<br>-.VFTDRNIEPPPDGQLK.-<br>-.GTPLITNHNYVITTK.-<br>-.LYLIGHELLK.-<br>-.DNLSSHTLEDLNNNWVK.-<br>-.VFEFIQQELNGKNELVK.-<br>-.NTIANLFVHPL.-<br>-.VINFDPTKDLESFVK.-<br>-.FVFIATYFDPSTDVLNNLPDNVK.-<br>-.FGIVVLATHVFFGATK.-<br>-.VFEFIQQELNGK.-	452	37.2(168/452)	5	271.10	27 (27 0 0 0 0)

ORF of MyunGV-A	Protein	Peptide Sequence	Length (aa)	Amino acid coverage (%)	Number of times detected in 29 samples	Sequest score (Delta Cn)	Peptides (Hits)
**14**	ODV-e56	-.QMDNIPDATINSLTTKK.-<br>-.VYNAPSGFITDHTQLIK.-<br>-.GNNGATSFDTIESCILR.-	353	14.4(51/353)	2	51.07	5 (5 0 0 0 0)
**16**	PEP	-.AYKELEEIDQKINK.-<br>-.AYKELEEIDQK.-<br>-.IYDLVVR.-<br>-.INKIYDLVVR.-<br>-.ALWKELEPEVNSEK.-<br>-.ELEPEVNSEKQFITSLGVR.-<br>-.YELSPAMHNLGNIFINEAIYDIR.-<br>-.ELEEIDQKINK.-	190	35.3(67/190)	21	160.49	16 (16 0 0 0 0)
**17**	PEP	-.DSLLCCISR.-<br>-.IFHLSPQALLCLPECEKR.-<br>-.ADAFANIFLTDVLCDLR.-<br>-.IFHLSPQALLCLPECEK.-<br>-.LYITALGVGLLACR.-<br>-.GCVVDNLATHDHSIPQR.-	153	49.0(75/153)	3	40.44	4 (4 0 0 0 0)

ORF of MyunGV-A	Protein	Peptide Sequence	Length (aa)	Amino acid coverage (%)	Number of times detected in 29 samples	Sequest score (Delta Cn)	Peptides (Hits)
**18**	PEP	-.TKLTNWAR.-<br>-.LWQDLAPGDVTFPPNK.-<br>-.ELLNILGHSMTHADEFPRSETK.-<br>-.VDGTDVPVFFSGVATDKPYVGVK.-<br>-.LIFSTRVDGTDVPVFFSGVATDKPYVGVK.-<br>-.LFTTEVGFAVYFGK.-<br>-.ELLNILGHSMTHADEFPR.-<br>-.MFDLIAQYIADPTPCNATNPLCMIPPGR.-	387	30.2(117/387)	22	910.30	91 (91 0 0 0 0)
**29**	Unknown	-.KVPQTSVVNLLDR.-	192	6.8(13/192)	1	10.82	1 (1 0 0 0 0)
**32**	Unknown	-.YHELQDKYDELR.-<br>-.FQYDRVQYEMK.-<br>-.FEHQLWPDLGPVTAPMTLNPFG.-	113	39.8(45/113)	1	70.99	7 (7 0 0 0 0)
**44**	Unknown	-.LLEELDDVVVEFLNKK.-	80	20(16/80)	1	10.53	1 (1 0 0 0 0)
**48**	Unknown	-.TLQRPLVDAVHIDALK.-	54	29.6(16/54)	1	10.41	1 (1 0 0 0 0)
**51**	ODV-ec43	-.YNSFNIHFPR.-<br>-.LYCEGDSLAISPGGVVPINRPTHNPK.-	353	10.2(36/353)	1	31.16	3 (3 0 0 0 0)
**64**	SOD	-.SIVVHAMEDDLGLGDNAQSK.-	153	13.1(20/153)	1	10.71	1 (1 0 0 0 0)

ORF of MyunGV-A	Protein	Peptide Sequence	Length (aa)	Amino acid coverage (%)	Number of times detected in 29 samples	Sequest score (Delta Cn)	Peptides (Hits)
**65**	Cath	-.NSWGSDWGENGYFR.-	338	4.1(14/338)	3	21.08	2 (2 0 0 0 0)
**67**	Unknown	-.QLYQSDYYGIGR.-<br>-.SLISPLFYVPSRR.-<br>-.MNPTYFAYGGVLR.-<br>-.SLISPLFYVPSR.-	56	67.9(38/56)	4	190.80	19 (19 0 0 0 0)
**103**	ODV-e25	-.GVEFSEIDSSNYMLR.-<br>-.FESNKMVYALVDASNSTLPELLR.-<br>-.DVSYPIVVLINNSSAQLVMK.-<br>-.VTVTETPLDYAGIIDNGNK.-<br>-.VSYGDSSIGKVTVTETPLDYAGIIDNGNK.-<br>-.AGANTVFLGILQNGLDGGATDSTSR.-	219	51.1(112/219)	5	161.03	16 (16 0 0 0 0)
**115**	p39	-.VDINQAPTGTR.-<br>-.SIACLNNDER.-<br>-.FTYIPQREPK.-<br>-.LSTGEYEGPIR.-<br>-.SNVDGTFICSYHLAR.-<br>-.EVFEIPSGVNNTSFK.-	327	49.8(163/327)	14	490.23	49 (49 0 0 0 0)

ORF of MyunGV-A	Protein	Peptide Sequence	Length (aa)	Amino acid coverage (%)	Number of times detected in 29 samples	Sequest score (Delta Cn)	Peptides (Hits)
		-.KEVFEIPSGVNNTSFK.-<br>-.YVFYTIYDEPDTVATQR.-<br>-.YLVGVSLIQQDTPEAYR.-<br>-.TFGDQNPESQTAFDAMPNFIK.-<br>-.FQVYNFVDFGGR.-<br>-.ITIPSKENYYSYLNVANMSSMEK.-					
**120**	Unknown	-.YLEGQINALR.-<br>-.MNHTNVHDLSPYR.-<br>-.KPFNNTYNSNVYVPPAILPAAPLYQFENR.-<br>-.VHKEILQQHK.-	123	50.4(62/123)	6	538.21	54 (53 1 0 0 0)
**125**	gp41	-.EVFISADPLPVTATK.-<br>-.RFETDDQLIDYYK.-<br>-.LNLASTHLSNVVK.-<br>-.YQMATAITQNKPLPIVQNDVADEYLK.-<br>-.FVSQTPQNSNQLYATELAFENEALR.-	290	31.7(92/290)	6	70.28	7 (7 0 0 0 0)
**156**	ODV-e66	-.FVYNNTQANQFK.-<br>-.LDDRFVYNNTQANQFK.-	667	16.3(109/667)	4	151.03	15 (15 0 0 0 0)

ORF of MyunGV-A	Protein	Peptide Sequence	Length (aa)	Amino acid coverage (%)	Number of times detected in 29 samples	Sequest score (Delta Cn)	Peptides (Hits)
		-.HINITNNPSFSNFAIR.-					
		-.GMFQLYDNILSIRPISNSAR.-					
		-.QGSNYSNVIGSFVSYK.-					
		-.MGLPYAYGQLLR.-					
		-.VRLEELNLAYDSYTFYHSK.-					
		-.QGSNYSNVIGSFVSYKNEAVSADFSK.-					
**157**	Enhancin-1	-.LIQNDSALFVR.-	828	14.7(122/828)	1	140.28	14 (14 0 0 0 0)
		-.LDIMGANTPLGR.-					
		-.LIHAQPENLYLLK.-					
		-.HVLNFVPGAVLR.-					
		-.AYPFSAALAYHKPSVYPVR.-					
		-.FLIDDYDTVSNNYDLK.-					
		-.LLEDHIEERPDSDFIIENINQAGR.-					
		-.NMYSEENQIYDTFLR.-					
**159**	Enhancin-3	-.VGENWIFAR.-	901	37.5(338/901)	16	1440.24	144 (144 0 0 0 0)
		-.MLDTALPSTQNIFAR.-					
		-.VIVPATVLPPWLR.-					

ORF of MyunGV-A	Protein	Peptide Sequence	Length (aa)	Amino acid coverage (%)	Number of times detected in 29 samples	Sequest score (Delta Cn)	Peptides (Hits)
		-.EQAAFLDNYSQLMYIENELR.-					
		-.DTNGAIVFSYSR.-					
		-.YPIKYIITDFDLVSK.-					
		-.QYLESNFDLVIPEELR.-					
		-.LIIFTWLYNPQR.-					
		-.EATIQALIDNNSPFDNWGFFER.-					
		-.TEVGVVLPANTK.-					
		-.RTEVGVVLPANTK.-					
		-.YKLHLAHSPR.-					
		-.VVHFNLHLR.-					
		-.AGFTRPVIVR.-					
		-.LHLAHSPR.-					
		-.ALALGQSVR.-					
		-.QTDLLADVR.-					
		-.LQLIVNK.-					
		-.ALALGQSVRYPIK.-					
		-.IANNHNGPHSYFDTLYFK.-					

ORF of MyunGV-A	Protein	Peptide Sequence	Length (aa)	Amino acid coverage (%)	Number of times detected in 29 samples	Sequest score (Delta Cn)	Peptides (Hits)
		-.QGYYKFDVVTYSSIR.-					
		-.YIITDFDLVSK.-					
		-.YIITDFDLVSKNYDIK.-					
		-.DSIYLASQLVDPASDEFVK.-					
		-.VVCVIDDPSQIVGEPFSVYDGNER.-					
		-.IANNHNGPHSYFDTLYFKVELR.-					
		-.ADAGGPGGAYYGPFWTAPASSNLGDYLR.<br>-					
		-.YVATIYFNPFEQTVTVHLNNIR.-					
		-.LREQAAFLDNYSQLMYIENELR.-					
**174**	Unknown	-.INTGNVYDDPEYQAK.-<br>-.ANNIAAIETVK.-<br>-.EIMTVVNQSVYR.-<br>-.ITTITLHTEIIR.-<br>-.LFTDIPYNQLNDK.-<br>-.TPADLFDNPLYQR.-<br>-.PTPDLNVPFIPK.-	373	42.9(160/373)	2	380.66	38 (38 0 0 0 0)

ORF of MyunGV-A	Protein	Peptide Sequence	Length (aa)	Amino acid coverage (%)	Number of times detected in 29 samples	Sequest score (Delta Cn)	Peptides (Hits)
		-.IINNMFTDKFNNAVFQHTYNR.-					
		-.LFTDIPYNQLNDKDFPNDEFR.-					
		-.TPADLFDNPLYQRPISNAVYIEK.-					
		-.TPADLFDNPLYQRPISNAVYIEKVNESSA.-					
		-.YYLLPRPTPDLNVPFIPK.-					
		-.LNVTLFQLLQESPGINAPLYK.-					
**175**	Unknown	-.ELITLPFKTPTVDTPVTQIYF.-	67	31.3(21/67)	1	30.67	3 (3 0 0 0 0)
